# Conductometric Sensor for Soot Mass Flow Detection in Exhausts of Internal Combustion Engines

**DOI:** 10.3390/s151128796

**Published:** 2015-11-13

**Authors:** Markus Feulner, Gunter Hagen, Andreas Müller, Andreas Schott, Christian Zöllner, Dieter Brüggemann, Ralf Moos

**Affiliations:** 1 Department of Functional Materials, Bayreuth Engine Research Center (BERC), University of Bayreuth, 95440 Bayreuth, Germany; E-Mails: Funktionsmaterialien@uni-bayreuth.de (M.F.); Funktionsmaterialien@uni-bayreuth.de (G.H.); Funktionsmaterialien@uni-bayreuth.de (A.M.); Funktionsmaterialien@uni-bayreuth.de (A.S.); 2 Department of Engineering Thermodynamics and Transport Processes, Bayreuth Engine Research Center (BERC), University of Bayreuth, 95440 Bayreuth, Germany; E-Mails: LTTT@uni-bayreuth.de (C.Z.); LTTT@uni-bayreuth.de (D.B.)

**Keywords:** on-board diagnostics (OBD), diesel particulate filter (DPF), soot sensor, accumulating sensor, soot-load determination, dosimeter

## Abstract

Soot sensors are required for on-board diagnostics (OBD) of automotive diesel particulate filters (DPF) to detect filter failures. Widely used for this purpose are conductometric sensors, measuring an electrical current or resistance between two electrodes. Soot particles deposit on the electrodes, which leads to an increase in current or decrease in resistance. If installed upstream of a DPF, the “engine-out” soot emissions can also be determined directly by soot sensors. Sensors were characterized in diesel engine real exhausts under varying operation conditions and with two different kinds of diesel fuel. The sensor signal was correlated to the actual soot mass and particle number, measured with an SMPS. Sensor data and soot analytics (SMPS) agreed very well, an impressing linear correlation in a double logarithmic representation was found. This behavior was even independent of the used engine settings or of the biodiesel content.

## 1. Introduction

In order to meet the increasingly tighter emission regulations, diesel particulate filters (DPF) have become serial standard in exhaust gas after treatment systems of diesel engines [1]. Soot particles generated during the combustion process are trapped in the pores of the walls of ceramic filter monoliths. Depending on the amount of stored soot on the filter, DPFs must be regenerated from time to time by soot oxidation to avoid clogging. An appropriate control strategy minimizes fuel consumption during regeneration and prevents the DPF from being damaged by high temperatures or thermal stress [2]. The state-of-the-art method is to indirectly determine the soot load based on soot load models, calibrated by the pressure drop over the DPF [3]. Since scales weighing is not possible in the exhaust pipe, no direct technique to evaluate the mass of stored soot is available. Instead, some recent research approaches suggest microwave-based techniques for that purpose [4,5,6].

Driven by on-board diagnostics (OBD) regulations to monitor DPF filtration efficiencies and tailpipe particulate matter (PM) emissions, recently, PM sensors have been developed to estimate excessive PM emissions downstream of a DPF in case of a filter failure [7,8,9,10,11,12,13,14,15]. In-use PM sensors are based on the conductometric principle. Driven by electrophoresis, soot particles deposit on the sensor surface and a current, *I*, occurs after a certain percolation time and continuously increases with more and more soot deposited on the sensor. This time span or the electrical current (respectively the resistance) at a certain time can be a measure for the amount of soot in the exhaust [8,9,10,11,12,13,14,15]. Some authors call this type of sensors “accumulating (or collecting) soot sensors” [7], since the occurring sensor current is a measure for the accumulated amount of soot on the sensor. Therefore, these sensors can also be classified as integrating devices that follow the discontinuously working dosimeter principle: during a “sorption” period, the sensor is able to measure, whereas soot has to be removed during a periodic “regeneration” phase [16] by burning off the soot. According to the dosimeter principle, it should be possible to obtain information on the actual concentration of a component by the first timely derivative of the sensor signal, if the sensor signal (here the current *I*) is proportional to the amount of sorbed species and the deposition rate is not a function of the amount of previously stored analyte (here soot) [14,16]. In the present contribution, we shall investigate whether such sensors are applicable for soot mass flow detection upstream of a DPF, *i.e.,* for high soot mass flows in non-filtered exhausts. The slope of the current *I* during sorption is the key parameter that should coincide with the amount of soot in the exhaust. This study, conducted on a dynamometer test bench, goes beyond initial results earlier suggested by Weigl *et al.* [13], who briefly introduced this principle for OBD purposes for low soot concentrations downstream of a DPF. It shall be pointed out here that the aim of the present study is to investigate whether this sensor principle can generally be applied also in automotive exhausts upstream of DPFs, where high soot concentrations occur. One application could be to integrate the measured soot mass concentration over time to determine the soot load of DPFs. Therefore, in this study, not the time span until a certain current threshold is reached, is considered here, but the timely derivative of the current, with the additional advantage that the signal is almost continuously available.

## 2. Experimental Section 

### 2.1. Sensor Setup 

**Figure 1:**
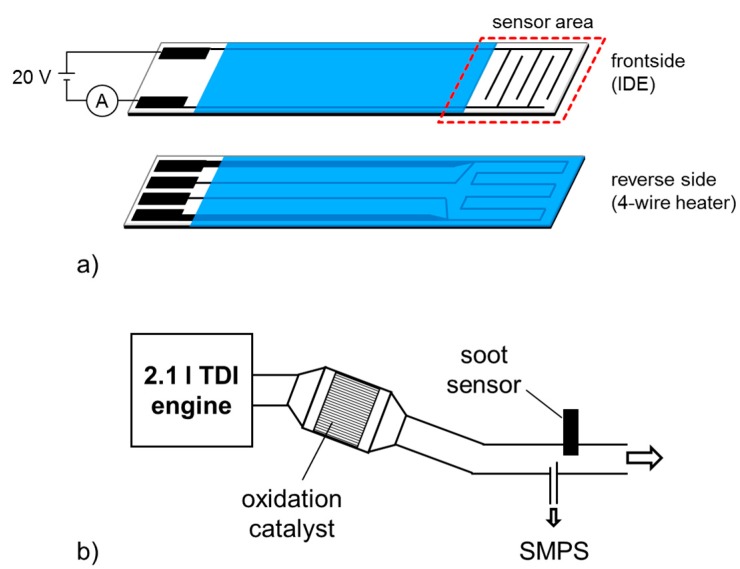
(**a**) Sensor design and (**b**) Dynamometer test setup and soot sensor mounting position.

Basically, conductometric soot sensors comprise (interdigital) electrodes on the top side of insulating ceramic substrates. On the reverse side, one usually finds heater structures. A typical setup of such a sensor is given in Figure 1a. A dc voltage is applied between the sensing electrodes, inducing an electrophoretic force, which attracts soot particles and hence leads to soot accumulation on the sensor surface. Due to the good electrical conductivity of soot, conductive paths form between the electrodes and an electrical current, *I*, results. With increasing soot accumulation on the sensor, also the current, *I*, increases [8,9,10,11,12,13]. The time-span until first conductive paths are formed out is sometimes called percolation time. To regenerate the sensor, the interdigital electrode area is heated up to several hundred °C to oxidize the soot. Regeneration is initiated after a certain current threshold is reached.

The here-used soot sensors as shown in Figure 1a, were built up in classical thick-film-technology on insulating alumina substrates (Rubalit 708S, CeramTec, Plochingen, Germany) as substrates for interdigital electrodes (IDE, line = space = 100 µm) and the heater structure. Both structures are made from Pt (LPA88, Heraeus, Hanau, Germany). The heater is designed in a 4-wire layout, which allows controlling the sensor temperature to a defined heater resistance in the sensor area. Electrical feed lines and the heater itself are covered (protection and insulation) by alumina based dielectrics (QM42, DuPont, Wilmington, DE, USA). 

After wiring, the sensor elements were housed in a simple stainless steel tube. At this stage, the heaters were calibrated by adjusting them to defined resistances and measuring the sensor temperature by a pyrometer (KT19II, Heitronics, Wiesbaden, Germany). One finds a linear correlation between heater resistance and temperature, which was taken later on to calculate the sensor temperature during soot deposition and to adjust the regeneration temperature during the regeneration phase.

Sensors were mounted in the exhaust pipe of a 2.1 l (2143 cm^3^) turbocharged direct injection (TDI) diesel engine, downstream of an oxidation catalyst, on a dynamometer test bench (Figure 1b). Thereby, the sensor was oriented in a way that the sensing IDE faced the gas flow directly.

### 2.2. General Sensor Characteristics 

**Figure 2:**
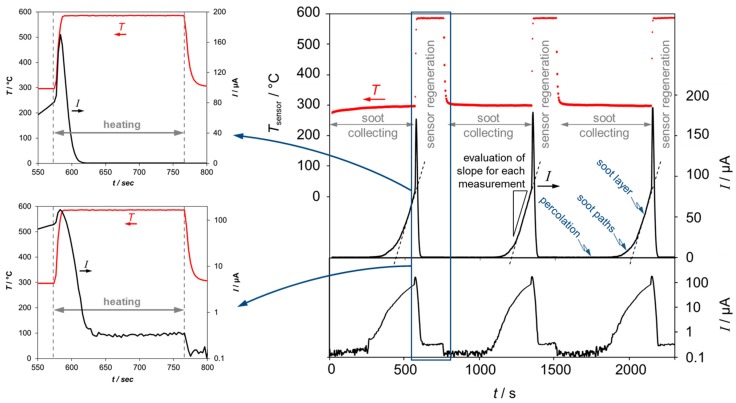
Typical raw data of the current *I* (at constant operation conditions) plotted linearly (right, upper graph) as well as logarithmically (right, lower graph). During soot collection, the sensor current *I* increases constantly. The evaluation of the current gradients (slope d*I*/d*t*) is a measure for the concentration of soot in the exhaust. Regeneration takes place by heating up the sensor to 600 °C. Left graphs: enlargements of the regeneration phase in linear and logarithmic representation. The small peak directly after heating stems from the increased soot conductivity with temperature before the soot is burned off.

During engine operation, the sensor signal was achieved discontinuously. By applying 20 V (DC) on the IDE, a current *I* appears (Figure 2) after a characteristic “percolation time”, which is the threshold when first soot paths are formed from one electrode to the other, e.g., [8,9,10]. The slope of this increasing current (d*I*/d*t*) was evaluated and considered as the sensor signal. After a distinct current value, a new cycle was started by heating the sensor to 600 °C to remove the previously deposited soot (regeneration phase). The regeneration takes about 1 min. Temperature was adjusted by a heater controller. After heating was switched off, the sensor cooled down to exhaust gas temperature and soot collection started again (sorption phase). In that phase, the heater (four-wire connection) was used as a temperature sensor by means of the above mentioned calibration curve. In Figure 2, this sensor temperature is plotted as well as typical data of the sensor signal *I* during constant engine operation conditions (boost pressure = 1.25 bar, injection pressure = 660 bar, *λ* = 1.26, exhaust temperature = 300 °C) in a linear (upper graph) and a logarithmic (lower graph) representation.

To evaluate a slope value that corresponds to a distinct soot emission at a certain engine operation point, we repeated at least three cycles of loading and regeneration of the sensor and calculated a mean value.

The applied voltage (here, 20 V DC) is required, as due to electrophoresis the soot particles are attracted and deposit on the surface of the sensor. During soot deposition three parts appear in the course of the current: 

(1) The percolation phase. Here the electrical current is almost zero, since during this phase, no conductive soot paths have been formed yet. 

(2) A path controlled period. When a soot path has formed between the electrodes, a current occurs, which increases almost monotonously the more paths between the fingers are built. 

(3) A thickness controlled phase, at which a more or less dense soot layer is formed. The current increases linearly, as the soot layer growths thicker. The slope of the sensor current is evaluated during the linear increase of this third phase (Figure 2).

A detailed view on the sensor signal during regeneration reveals the following. When starting the regeneration, *i.e.*, when switching on the heater, the sensor signal initially increases. After a certain time, the current decreases rapidly but remains in the measurable µA-range as long as the sensor temperature is set to the regeneration temperature. When the heater is switched off and the sensor temperature decreases to exhaust temperature, the current decreases to almost zero. This behavior becomes particularly obvious in the logarithmic representation (lower graph) of Figure 2. These results agree with earlier findings evaluating the electrical resistance between soot sensing electrodes [8]. The increasing current in the beginning of the regeneration procedure is attributed to the negative temperature coefficient of resistance of soot [17]. Not before soot oxidation starts, the current starts to decrease. The non-zero current at 600 °C is not an effect resulting from soot, but is owing to the conductivity of the alumina substrate, which cannot be considered anymore as an ideal insulator at these high temperatures [18]. Therefore, for each single sensor substrate, the current value at 600 °C depends only on the electrode structure, *i.e.*, on the IDE line and space width. This value may be used for sensor OBD purposes, since one could see defects of single fingers at each regeneration procedure.

### 2.3. Engine Parameters and Analytics 

For a wide soot concentration variation, engine parameters like boost pressure and/or injection pressure were varied. Speed and accelerator position were kept constant (1000 rpm/25% accelerator position) to avoid too large differences in mass flow and to keep the results comparable. Fuels with different biodiesel contents were used. “B7” stands for 7% and “B100” for 100% biodiesel content.

The soot concentration was measured simultaneously by SMPS (electrostatic classifier: TSI 3080 with long DMA 3081, CPC: TSI 3022A, TSI Inc., Shoreview, MN, USA). The soot was sampled near the sensor mounting position (Figure 1b) with two dilution steps (1:36 and 1:11) and an intermediary evaporation tube (350 °C). The obtained SMPS results (particle size distribution) were processed to calculate the volumetric soot mass and the particle concentrations, assuming spherical particles with a density of 1.7 g/cm^3^. By means of the exhaust mass flow, which was calculated from the intake air, from the injected fuel mass, and from an assumed density of the raw exhaust gas of 1.3 kg/m^3^ at 273.15 K (corrected to the actual temperature), the volumetric exhaust flow rate was estimated for each run to derive a particle mass and particle number per hour.

**Figure 3:**
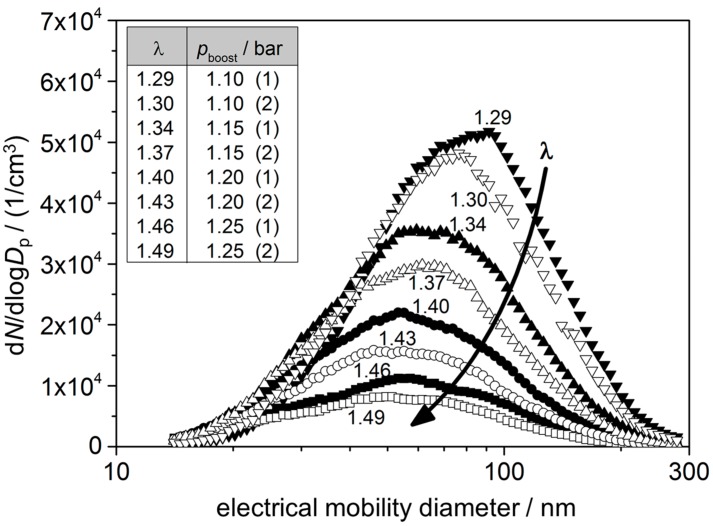
Result of soot analytics (SMPS) measurements for different operation points at 1000 rpm/25% accelerator position with B100 fuel. Only the boost pressure was changed (*p*_injection_ = const.), the other parameters are depended according to the engine characteristic map. Inset: correlation between boost pressure and resulting lambda values (the numbers in brackets indicate the first or second measurement run).

In our investigations, we varied the parameters “boost pressure” (*p*_boost_) and “injection pressure” (*p*_injection_). All other parameters were adjusted automatically in accordance to the engine characteristic map. Neither the setup nor the intake air was conditioned. For each operating point, characterized by boost and injection pressure at constant speed and accelerator position, a certain *λ* value (lambda, air–fuel equivalence ratio) occurred. Here, we found good correlations - despite of the above mentioned non-ideal setup without intake-air-conditioning. The soot generation for each condition was analyzed by SMPS (end of pipe) as particle size and number distribution. As expected, we found an increasing amount of soot and overall larger soot particles with decreasing *λ* values. Figure 3 gives an exemplary overview on such a dependency for selected conditions with B100.

As can be obtained from Figure 3, *λ* increases with the boost pressure, leading to a decreasing soot generation. This behavior was expected and can be explained by conditions that become more and more ideal for the combustion process with increasing boost pressure.

**Figure 4:**
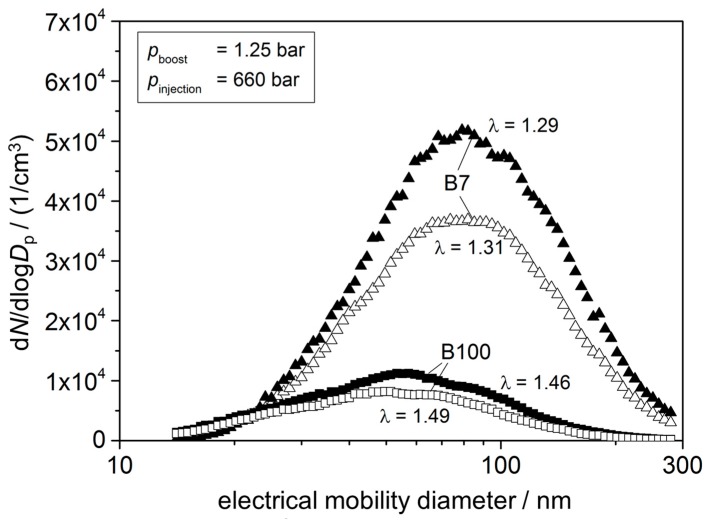
Result of SMPS measurements for different biodiesel content of 7% and 100% at similar engine operation points (1000 rpm; 25% accelerator position; *p*_boost_ = 1.25 bar; *p*_injection_ = 660 bar).

Taking other fuels into account, higher lambda values could be expected for increasing biodiesel contents, leading to a decreasing soot generation. Since biodiesel contains more oxygen atoms in its chemical structure, the combustion process should be enhanced [19]. If one regards data achieved in similar operation points (*p*_boost_ = 1.25 bar, *p*_injection_ = 660 bar), a significant dependency of the soot generation on the used fuel occurs (Figure 4). Here again, varying *λ* values are found. The correlation goes along with findings before: higher lambda values correspond to less soot generation. Lowest soot values are reached with 100% biodiesel operation. Variations in soot generation within the measurements with same diesel fuel are due to the non-ideal setup.

In summary, there is a wide range of adjustable amounts of soot by changing boost and injection pressure and using different kinds of diesel fuel. Note here again that all investigations were conducted at constant engine speed and load.

## 3. Results

Now, the sensor data (for each characteristic operation point, we measured the sensor signal, *i.e.*, the slope of the current, for at least three times) are correlated with the soot content in the exhaust. Altogether, a good and reproducible correlation can be observed, but a strong impact of the sensor orientation with respect to the exhaust flow direction affects the results. As expected, the signals were higher when soot is directly deposited on the measuring electrodes. Furthermore, spreading of the evaluated data is less in the direct mode (*i.e.*, with sensing IDE facing directly the gas flow; for further details see [18]). As a consequence, the test runs were conducted in the direct mode due to the higher signals and a faster soot loading especially at low soot concentrations.

**Figure 5:**
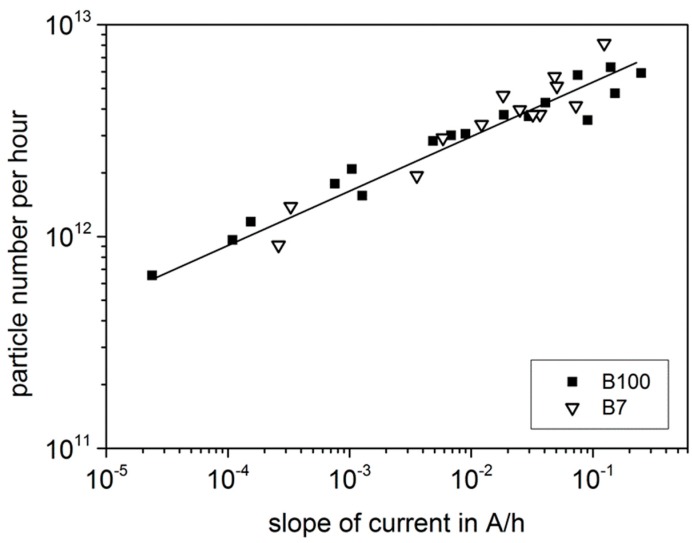
Correlation between evaluated sensor data and achieved particle number per hour.

In the next figures, the correlation of the sensor signal (slope d*I*/d*t* in A/h) and the soot particle flow (in particles/h) or the soot mass flow (in mg/h) are shown. The “particle number per hour” was evaluated from SMPS data as described above, taking into account the number concentration, the exhaust mass flow and the exhaust density, corrected with temperature. As can be seen in Figure 5, the sensor signal increases with increasing particle number in the exhaust flow. In the double-logarithmic representation, a linear dependency with a small scatter band occurs over almost 4.5 decades of the sensor signal.

**Figure 6:**
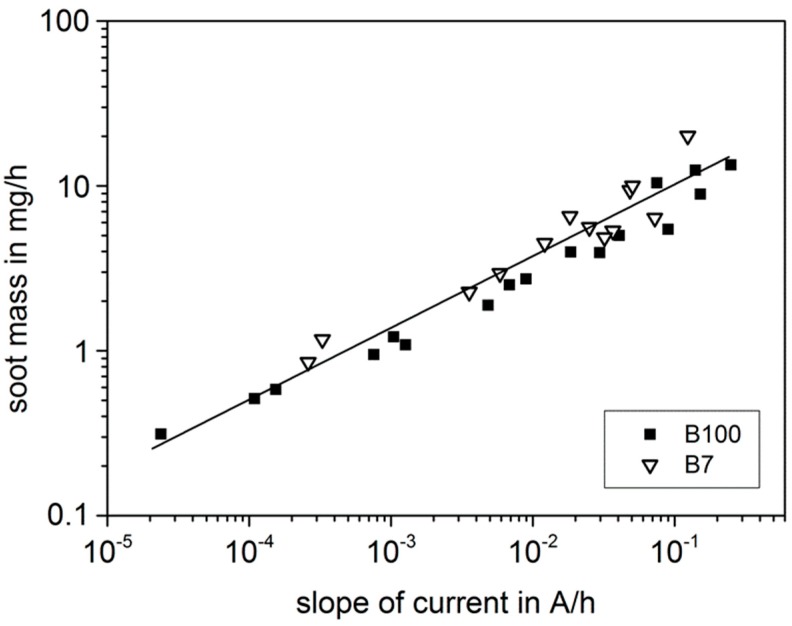
Correlation between evaluated sensor data and achieved particle mass per hour.

Similar results are achieved for the evaluation with respect to the particle mass (Figure 6). Here, the double-logarithmic representation reveals an impressing linear dependency, independent on the used fuel.

The difference between particle mass and number mirrors the SMPS data. The position of the maximum of the particle size distribution can be roughly seen as an indication for the medium particle size, whereas the integral product of size and number represents the soot mass. It is clear that number and overall soot mass are basically independent, as the exhaust gas can, e.g., contain a huge number of very small particles or only few large particles.

Both parameters, the soot mass and the particle number per hour, describe the soot raw emission and that contributes to the soot-load of the DPF. The sensor signal directly depends on the soot concentration, respectively the soot mass flow, regardless which type of fuel or which parameter setting is used to achieve this amount of soot. On the sensing electrodes, a soot layer of deposited soot particles is formed. With increasing thickness, the current increases (at constant voltage). As we measure in the raw exhaust (not downstream a DPF as introduced in [9]), we cannot assume only single soot paths contributing to the sensor signal. Owing to the high soot concentration, very shortly after the first soot path has been formed, a complete layer covers the sensor electrode. The increasing layer-thickness results in an increasing current. Furthermore, downstream of a DPF, only very small soot particles should be present in the exhaust, which are attracted of the electric field in a way that they form “dendrite-like” paths between the fingers of the interdigital electrodes. Here in our application, the particles are too huge to form such growing paths. 

## 4. Conclusions and Outlook

The present investigations should prove whether a conductometric soot sensor, similar as it is today’s standard for DPF-OBD, is applicable for soot mass determination upstream of a DPF. Sensor data were achieved in dynamometer tests in real exhausts of a diesel engine by evaluating the slope of the increasing current during soot deposition on top of interdigital electrodes. The amount of soot was varied by changing the boost or injection pressure in one constant operation point (1000 rpm/25% accelerator position). The sensor results are direct results: the signal depends directly on the soot in the exhaust gas flow with respect to both particle number and particle mass.

We found a very good correlation between sensor data and simultaneous soot analysis by SMPS. The slope of the electrical current increases with increasing soot mass or particle number per time interval. In the double-logarithmic representation, a linear dependency occurs over a wide range of 4.5 decades of current slope which corresponds to 2 decades of soot mass concentration. Mounted upstream of a DPF, the sensor can be theoretically used for soot load determination of a DPF by integrating the soot mass.

In further measurements one topic seems to be relevant which relates to the operating method of the sensor. Higher voltages applied at the electrodes should “collect” more or different sized soot particles [20,21]. We will prove this and also correlate the sensor signal with defined soot particle number and mass. Variations for these investigations will be conducted in a lab setup comprising a soot generator. The orientation of the sensor electrodes (either facing the gas flow or facing the muffler) plays an important role for the interpretation of the sensor results [22] and will be addressed in future studies. For an application, it is crucial to focus on the development of an appropriate sensor housing including a protection cap, ensuring constant flow conditions on the sensors surface. Another topic of future research will be to investigate whether these results and a contactless method for in-situ soot loading detection of a DPF [8,11] coincide. Furthermore, different sensor designs concerning the dimensions of the IDE might improve sensitivity or allow for measuring particulate matter of specific size. Adding a thin cover layer on top of the sensing-electrodes enables soot detection by measuring the capacitance between the electrodes, which changes when soot accumulates on the sensor tip [23,24]. Additionally, further investigations of the influence of different engine operating conditions on the soot sensor signal are necessary to study different soot characteristics [25,26,27,28] and exhaust gas properties, like, e.g., mass flow or temperature.
